# Commercial amniotic membrane extract eye drops for canine SCCED: a retrospective clinical evaluation

**DOI:** 10.3389/fvets.2025.1658719

**Published:** 2025-08-13

**Authors:** Jing Wen Luo, Zhen Lei Zhou

**Affiliations:** ^1^College of Veterinary Medicine, Nanjing Agricultural University, Nanjing, Jiangsu, China; ^2^Focus Pet Hospital, WuXi, Jiangsu, China

**Keywords:** amniotic membrane extract, corneal defects, corneal erosion, ocular surface disease, ophthalmic solution

## Abstract

**Objectives:**

This study aims to evaluate the efficacy of amniotic membrane extract eye drops (AMEED) in promoting the healing rate of SCCED after cotton-tip debridement (CTD) in dogs.

**Methods:**

A retrospective review was conducted on the medical records of dogs with SCCED from October 2021 to December 2024. Cases treated with AMEED following CTD (AMEED group), as well as those treated with CTD alone (CTD group), were selected for further evaluation. Data analyzed included age, sex, breed, medical therapies and procedures used, time to healing, performance of a second procedure, and complications.

**Results:**

A total number of 20 eyes from 18 dogs were included in the analysis. Eleven eyes of 10 dogs were in the CTD group, whereas nine eyes of eight dogs were in the AMEED group. The healing rates were 27.2% (3/11) in the CTD group and 77.8% (7/9) in the AMEED group following a single CTD procedure. The mean time of healing following a single CTD procedure was 16.6 days (range: 11–21 days) in the AMEED group and 10.6 days (range: 7–14 days) in the CTD group, respectively. The longer healing time in the AMEED group may be due to more chronic cases and the presence of corneal granulation tissue compared to the CTD group. A second procedure was performed on two eyes in the AMEED group and five eyes in the CTD group that failed to heal after one procedure. For the eyes that healed in both groups, no complications were observed.

**Conclusion:**

Topical therapy with AMEED is a safe and effective adjuvant treatment for canine SCCED. AMEED may be preferred in situations where more invasive procedures are less favorable.

## 1 Introduction

Spontaneous chronic corneal epithelial defects (SCCED) represent a common and refractory corneal ulcer in dogs. Dogs affected with SCCED commonly display clinical signs that persist for several weeks to months prior to being referred for veterinary intervention (1, 2). The average age of onset for this disease is between 8 and 9 years (1, 3). The clinical manifestation of this chronic non-healing corneal ulcer is characterized by the presence of a nonadherent epithelial lip surrounding superficial, non-infected corneal defect (3). The specific pathogenesis underlying SCCED remains to be elucidated. Histopathological evaluation in canine patients with SCCED often reveal dysmaturation of the poorly attached epithelial lip, a thin superficial stromal hyaline acellular zone (HAZ), an abnormalities in the basement membrane (BM) structure, and the alterations in corneal innervation at the site of erosion (1, 4, 5). Currently, surgical therapies aimed at facilitating the regeneration of a robustly adhered epithelial surface are regarded as first-line treatment option for SCCED (1). Common surgical treatments include epithelial debridement, punch or grid keratotomy, and superficial keratectomy. However, these surgical procedures may result in additional corneal trauma and exacerbate discomfort in affected animals. Interestingly, the clinical presentations and histopathological features of SCCED in dogs closely resemble those observed in persistent corneal epithelial defects (PED) caused by recurrent epithelial erosions in humans (4, 6, 7). Moreover, an investigation into the genetic defect of Boxer dogs with SCCED has indicated that a deficiency in the Noggin (NOG) gene may contribute to both SCCED and recurrent epithelial erosions (8). In human ophthalmology, amniotic membrane (AM) has long been an effective therapeutic option for managing PED (9, 10). We postulate that AM may similarly facilitate the treatment of SCCED in dogs.

The amniotic membrane has the functions of inhibiting inflammation, resisting fibrosis, and suppressing angiogenesis, and also helps in the proliferation of epithelial cells (11–13). The ability of AM to facilitate corneal wound healing is attributed to its rich content of various growth factors (6, 10, 14). Traditionally, AM is applied in ophthalmology as a fresh or cryopreserved graft for the reconstruction of corneal defects. A recent study indicated that the clinical efficacy of sterile lyophilized amniotic membrane extract (AME) was superior to that of AM transplantation in a corneal wound model (11). Except for the structural support property, AME is rich in therapeutic components essential for corneal repair, similar to frozen AM (11, 13, 15, 16). A significant advantage of amniotic membrane extract eye drops (AMEED) compared to AM transplantation lies in its ability to continuously deliver bioactive growth factors to the injured cornea throughout the entire treatment period, eliminating the need for invasive intervention (10).

Recent studies have investigated the efficacy of commercial AMEED in promoting the healing of experimental superficial corneal ulcers in rats, cats, and horses (14, 17, 18). These reports have confirmed the safety of commercial AMEED for this application. However, the data revealed that its application does not reduce re-epithelialization time of experimental superficial corneal ulcers or improve the healing efficiency of these ulcers. The findings derived from experimental studies may not align with clinical observation (18). Human clinical investigations have revealed a strong correlation between the therapeutic efficacy of amniotic membrane and the specific ocular diseases it targets (19, 20). Sabater-Cruz et al. pointed out that AMEED demonstrated significant efficacy in clinical applications for treating persistent epithelial defects in humans (21). Therefore, the purpose of the present study is to evaluate the efficacy of commercial AMEED as an adjuvant to CTD in dogs with SCCED.

## 2 Materials and methods

### 2.1 Animals, ophthalmic examination, inclusion criteria, and exclusion criteria

Canine patients with spontaneous chronic corneal epithelial defects (SCCED) at our clinic were enrolled in this retrospective study. Data was reviewed from October 2021 to October 2024. All patients were client-owned dogs. Ophthalmic examinations were performed by the same attending clinician. All dogs underwent a set of ophthalmic examination which included slit-lamp biomicroscopy (SL-17, Kowa, Kowa Life Science Division, Japan), fluorescein staining (I-DEW FLO, Entod Research Cell UK Ltd., United Kingdom), Schirmer tear test I (Eickemeyer^®^, Tuttlingen, Germany), and rebound tonometry (Tonovet, Icare Finland, Oy, Finland). The diagnosis of SCCED was established based on its specific clinical manifestations: a superficial corneal ulcer with non-adherent surrounding epithelium, a halo-fluorescein dye staining pattern, absence of signs indicating infection, and no identifiable physical pathogenic factors (e.g., ocular foreign body or eyelid margin abnormality). Only patients with SCCED’s clinical signs lasting more than 2 weeks prior to presentation and having at least two follow-up rechecks after being treated with AMEED or CTD alone were eligible for inclusion. The exclusion criteria include patients with concurrent ocular or systemic diseases that may interfere with the healing process, such as keratoconjunctivitis sicca, endothelial degeneration, diabetes mellitus, immune-mediated disease, etc.

Information harvested from the medical records included age, breed, sex, affected eye, prior medical therapies, procedure performed prior to presentation, signs duration of SCCED prior to presentation, concurrent corneal granulation tissue, therapeutic drugs, time to healing, need for a second surgical procedure, and any discomforts during AMEED treatment. Patients treated with AMEED following CTD were categorized into the AMEED group, while those treated with CTD alone were assigned to the CTD group. Patients who presented with a SCCED in the contralateral eye during a separate visit were classified as new cases. Table 1 presents an overview of the patients enrolled in this study.

**Table 1:** An outline of study cases.

Case	Breed	Age (years)	Sex	Eye affected	Procedure performed prior to presentation	Corneal granulation tissue at presentation	Signs duration of SCCED prior to presentation (days)	Success after one treatment	Time to healing following one treatment(days)	Procedure performed following the failure of the first treatment
AMEED group										
1	Chinese rural dog	8	F	OD	No	No	17	Yes	13	–
2	Teddy	8	M	OD	Two CTDs	Yes	36	Yes	15	–
3	Chihuahua	15	F	OD	No	No	27	Yes	21	–
4	Teddy	9	M	OS	No	Yes	96	Yes	11	–
5	Teddy	10	M	OD	No	No	67	Yes	14	–
6	Samoyed	14	F	OS	No	Yes	180	No	–	CTD + GK
7	Shiba Inu	8	M	OD	No	No	22	No	–	CTD + GK
8	Samoyed	10	M	OS	CTD + GK + BCL	Yes	180	Yes	21	–
9	Poodle	13	M	OD	No	No	90	Yes	21	–
CTD group										
1	Teddy	12	M	OD	CTD	Yes	30	No	–	CTD + GK
2	Chihuahua	7	M	OD	No	No	36	Yes	11	–
3	Teddy	7	M	OS	No	No	18	Yes	7	–
4	Teddy	10	M	OD	No	No	30	No	–	Lost
5	French bulldog	4	M	OD	No	No	21	No	–	Lost
6	Teddy	13	M	OS	No	No	60	No	-	CTD + GK
7	Pomeranian	11	F	OS	No	No	14	Yes	14	–
8	Shiba Inu	3	M	OS	No	Yes	60	No	–	CTD + GK
9	Chinese rural dog	10	M	OS	No	No	30	No	–	CTD + GK
10	Samoyed	9	M	OD	No	No	14	No	–	lost
11	Golden retriever	9	M	OS	No	No	30	No	–	CTD + GK

M, male; F, female; OD, right eye; OS, left eye; SCCED, spontaneous chronic corneal epithelial defects; CTD, cotton-tip debridement; AMEED, amniotic membrane extract eye drops; GK, grid keratotomy; BCL, bandage contact lens.

### 2.2 Treatment regime

All patients received the same protocol and dosage frequency to ensure standardization. After diagnosis confirmation, topical anesthesia was achieved with several drops of proparacaine hydrochloride (Alcaine^®^, 0.5% proparacaine hydrochloride eye drops, s. a. Alcon-Couvreur n. v., Belgium). The dogs were gently restrained manually. The non-adherent epithelium was removed with a CTD until stable epithelium was reached. In the CTD group, the topical treatment included autologous serum (AS), antibiotic, and hyaluronate acid (HA). In the AMEED group, all patients received similar topical medicines to those in the CTD group and an additional treatment with AMEED (EyeQ^®^ Amniotic Eye Drops, Vetrix^®^, Cumming, GA, United States). The topical antibiotic eye drops administered to each dog was one of chloramphenicol 0.25% drops (Runshu, Bausch+Lomb, Shandong, China), tobramycin 0.3% drops (Tobrex^®^, S. A. Alcon-Couvreur N. V., Belgium), or levofloxacin 0.5% drops (Santen Pharmaceutical Co., Ltd., Japan). A 0.75% solution of crosslinked hyaluronic acid (X-HA) (Oculenis Biohance^®^, SentrX Animal Care, Inc., Salt Lake City, UT, United States) was administered to each dog. In the AMEED group, AMEED was applied 15 min after the administration of the antibiotic eye drops. Furthermore, HA was instilled 30 min after the application of AMEED. In both groups, topical medicines were administered four times daily until no fluorescein uptake was observed during the re-evaluations. Additionally, oral meloxicam (Shanghai Hanwei Biomedical Technology Co., Ltd., China) was administered at a dosage of 0.1 mg/kg once daily for 3–5 consecutive days to all dogs.

### 2.3 Follow-up

The initial follow-up examination was recommended for patients 7 days after the treatment in both groups. When the corneal ulcer showed obvious evidence of healing such as a reduction in the size of the epithelial defect at the time of reevaluation, a second recheck examination was recommended 7 days later. If the SCCED did not exhibit clear signs of healing after 2 weeks of treatment—such as a persistent epithelial defect that remained the same size or increased in size—an additional intervention (CTD + GK with BCL) was implemented. During the entire treatment period, patients were advised to continue wearing the Elizabethan collar to prevent self-trauma. The SCCED was considered resolved after the recheck examination revealed a fluorescein-negative corneal surface. In the AMEED group, owners were instructed to observe and record any signs of ocular discomfort, such as blinking or conjunctival congestion, during the administration of the treatment. These observations were documented at the subsequent follow-up visit. Since corneal scarring affects both visual function and cosmetic appearance, the cornea opacity was evaluated for each case during a three-month observation period following complete healing of the cornea.

## 3 Results

### 3.1 Patients

A total of 20 eyes (18 dogs) were included in this study. One dog in each group was affected in both eyes. The average age at presentation was 10.5 years (range: 8–15 years) in the AMEED group and 8.6 years (range: 3–13 years) in the CTD group, respectively. Male dogs were observed more frequently than females in both groups. All cases in both groups had received prior antibiotic treatment. Prior to referral to our clinic, two cases in the AMEED group and one case in the CTD group had undergone procedures, all of which were conducted at least 2 weeks before the referral. The average duration of clinical symptoms before presentation was 79.4 days (range 17–180 days) in the AMEED group and 31.8 days (range: 14–66 years) in the CTD group, respectively. Corneal granulation tissue was observed in four of the nine eyes in the AMEED group, whereas none of the 11 eyes in the CTD group exhibited such sign at the time of initial presentation.

### 3.2 Clinical results

**Figure 1:**
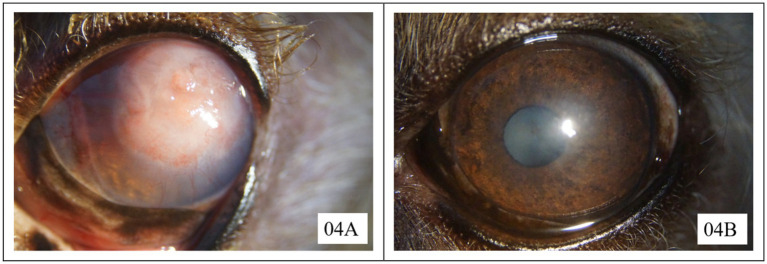
Corneal transparency of case 4 in the AMEED group at the initial presentation **(A)** and 3 months after complete healing **(B)**. Neither of the photographs was captured under fluorescein staining.

Only one eye in the CTD group was healed at the first recheck. At the second recheck, four eyes in the AMEED group and two eyes in the CTD group were observed to have healed. The eyes that had not fully healed by the second recheck were allowed additional time for recovery. Two cases in the AMEED group and five cases in the CTD group, which exhibited no signs of improvement following a 14-day course of treatment, subsequently underwent a CTD + GK with BCL procedure. Healing was confirmed in all seven cases 14 days after the second procedure. Three cases in the CTD group declined the additional procedure and were lost to follow-up. Finally, successful healing was observed in seven out of nine eyes (77.8% [7/9]) in the AMEED group and three out of 11 eyes (27.2% [3/11]) in the CTD group. The mean time to healing after a single treatment was 16.6 days (range: 11–21 days) in the AMEED group, compared to 10.6 days (range: 7–14 days) in the CTD group. Upon the detection of complete healing, the eyes exhibited either no scarring or only mild to moderate scarring in both groups. Three months after complete healing, despite initially presenting with the most severe granulation tissue in this case series, the cornea of case 4 in the AMEED group achieved excellent transparency, as shown in Figure 1. The other corneas also exhibited no or mild opacity during the three-month observation period following their healing. In the AMEED group, no cases of discomfort were observed following the administration of AMEED.

## 4 Discussion

This study evaluated the outcomes of commercial AMEED as a simple, convenient, and effective medical treatment for SCCED in dogs. Clinical data indicate that SCCED is clinically recognized as the most prevalent type of corneal ulcers in dogs (22–24). However, a universally accepted standard treatment protocol for this condition remains lacking. Medical therapies previously reported before 2000, such as topical polysulfated glycosaminoglycans, aprotinin, and epithelial growth factor, are currently rarely mentioned in contemporary discussion regarding the treatment of SCCED in dogs (25–27). Since 2000, additional medical therapies have been explored, including topical substance P, insulin-like growth factor, combinations of topical antimicrobial-chondroitin sulfate, aminocaproic acid, cyanoacrylate tissue adhesive, heterologous serum, autologous plasma, autologous platelet-rich plasma (PRP), a regenerative agent called “Cacicol,” and a natural eye drop known as Vizoovet^®^ (4, 28–34). In the realm of surgical interventions, various techniques have been attempted, including cotton-tip debridement (CTD), diamond burr debridement (DBD), scalpel blade debridement (SBD), punctate keratotomy (PK), grid keratotomy (GK), corneal thermal cautery (CTC), and superficial keratectomy (SK) (1–3, 33–46). These surgical procedures are frequently combined with the application of a bandage contact lens (BCL) to optimize healing conditions. Some studies have employed combined therapies, integrating medications and/or surgical approaches to enhance patient comfort while minimizing the duration of wound healing. The primary aim of these treatments is to maximize the healing rate by combining one or more methods, while minimizing complications (45).

Mechanical procedures addressing the HAZ in the anterior stroma are critical steps in the treatment of SCCED (5, 38, 39, 45). Various techniques for debridement have been described, including CTD, DBD, and SBD. Despite its simplicity and low cost, the debridement efficacy of CTD is relatively lower compared to that of DBD and SBD (2, 47). However, there is a potential risk of developing infectious keratitis following DBD (1). The selection of CTD in the current study was primarily driven by its simplicity and convenience. According to previous reports, the healing rate after a single CTD was found to range from 39 to 53.6% (4, 28–31). In the present study, a single-treatment healing rate of SCCED in the CTD group was recorded at 27.2%, which is lower than that reported in previous studies. In contrast, a healing rate of 77.8% for SCCED in the AMEED group was observed after a single CTD in the present study. This further suggests that the therapeutic efficacy of CTD alone is relatively limited. According to previous studies, the average healing time for most treatments was around 2 weeks. In the present study, the average healing time in the AMEED group was 16.6 days, compared to a shorter average of 10.6 days in the CTD group. This discrepancy may be explained by the longer duration of clinical symptoms before presentation, as well as the relatively higher incidence of corneal granulation tissue observed in the AMEED group. Although the exact time for complete healing cannot be determined due to the timing of re-evaluation, the post-treatment corneal healing time in the present study is comparable to that of previous treatments. Furthermore, AMEED is easy to use and does not cause additional corneal damage or increase animal discomfort. Overall, the therapeutic efficacy of commercial AMEED is comparable to that of conventional treatments previously employed.

Numerous experimental and clinical studies have demonstrated the efficacy of AME in promoting corneal epithelial cell proliferation, preventing stromal keratocyte loss, inhibiting inflammation, and accelerating collagen remodeling (10, 11, 13, 18, 48–50). Several mechanisms have been proposed to elucidate the role of AME in promoting corneal epithelialization and healing. Firstly, AME contains a variety of growth factors (11). These growth factor components in AME resemble those found in tears, which play a crucial role in corneal repair. Multiple factors synergistically facilitate the migration and proliferation process of corneal epithelial cells (50). Secondly, AME induces the proliferation of limbal stem cells, expedites the regular healing process of corneal epithelium, and preserves the population of normal pre-stromal keratocyte cells (11, 13). Additionally, AME also aids in restoring damaged corneal epithelial cells from an inflammatory state (49). Moreover, although rarely mentioned in the explanation of AM’s mechanism before, it is worth noting that AM also has the effect of accelerating nerve repair and enhancing nerve function (51). As early as 2001, Murphy et al. proposed that dogs with SCCED exhibited significant alterations in corneal innervation, characterized by disorganized areas of hyperinnervation in the subepithelial stroma and in the surrounding epithelial regions (4). The authors hypothesize that these underlying mechanisms may potentially elucidate the efficacy of AME in facilitating the restoration of the diseased corneal epithelium in dogs with SCCED, thereby enabling a microenvironment conducive to physiological healing processes. However, further investigations are warranted to confirm these findings.

This study presents a practical clinical case application in which the treatment regimen involved the combination of AMEED, antibiotics, autologous serum, and 0.75% X-HA. Therefore, caution should be exercised when interpreting the clinical efficacy of AMEED. The combined treatment regimen in the present study may account for potential variations in results compared to previous studies conducted on rats, cats, and horses. In a comparative study evaluating AS, AME, and artificial tears on alkaline corneal epithelial wounds in a rabbit model, it was indicated that AME exhibited superior efficacy in accelerating corneal epithelial healing compared to AS and artificial tears (52). A study conducted by Goncalves e Souza et al. demonstrated that 0.75% X-HA exhibited superior efficacy in promoting the healing of corneal epithelium compared to AMEED in a rat model (17). However, in the study conducted by Williams et al. in 2017, none of the five dog eyes with superficial epithelial erosion achieved successful healing when treated with 0.75% X-HA (53). Therefore, it is postulated that X-HA may not effectively facilitate the healing process of SCCED. In recent years, Scheffer C. G. Tseng’s team has conducted extensive studies on a novel matrix component called heavy chain (HC)-hyaluronan (HA)/pentraxin 3 (PTX3) found in the amniotic membrane. Their studies found that HC-HA/PTX3 exhibits potent anti-inflammatory, anti-scarring, and anti-angiogenic properties, serving as a pivotal constituent responsible for the biological functionality of AM (54). The exact covalent linkage between X-HA and the constituents in AM which leads to the formation of HC-HA or HC-HA/PTX3 as active agents remains uncertain. In the present study, all eyes in the AMEED group were administered a combination of 0.75% X-HA and AMEED. Therefore, it is imperative to acknowledge the potential existence of an interaction between X-HA and AM, which may ultimately facilitate the healing process of SCCED.

The study conducted by Levitt et al. in 2020 revealed that the presence of bacteria on the corneal surface of SCCED could be managed without significantly impacting recovery outcomes (55). Therefore, this study did not pay much attention to the bacterial cultures. None of the patients in the AMEED group showed signs of increased tearing, conjunctival congestion, or blepharospasm following the application of AMEED. The primary limitation of the current study is its small sample size. Additionally, there are inherent limitations associated with retrospective studies that may affect the generalizability of the findings.

## 5 Conclusion

Amniotic membrane extract eye drops exhibit effective therapeutic potential for the treatment of SCCED in dogs, offering the significant advantage of eliminating the need for general anesthesia or specialized surgical skills. Considering the pathophysiological similarities between SCCED and recurrent corneal epithelial erosions in humans, the clinical application observed in veterinary medicine may provide valuable insight for the treatment of such conditions in the human cornea. Further research via prospective randomized trials is required to validate these findings.

## Data Availability

The original contributions presented in the study are included in the article/supplementary material, further inquiries can be directed to the corresponding author.
